# Involvement of the conserved *Hox *gene *Antennapedia *in the development and evolution of a novel trait

**DOI:** 10.1186/2041-9139-2-9

**Published:** 2011-04-19

**Authors:** Suzanne V Saenko, Marta SP Marialva, Patrícia Beldade

**Affiliations:** 1 Institute of Biology, Leiden University, Sylviusweg 72, 2333 BE Leiden, The Netherlands; 2 Current Address: Department of Genetics and Evolution, University of Geneva, 1211 Geneva 4, Switzerland; 3 Instituto Gulbenkian de Ciência, Rua da Quinta Grande 6, P-2780-156 Oeiras, Portugal

## Abstract

**Background:**

Hox proteins specify segment identity during embryogenesis and have typical associated expression patterns. Changes in embryonic expression and activity of *Hox *genes were crucial in the evolution of animal body plans, but their role in the post-embryonic development of lineage-specific traits remains largely unexplored. Here, we focus on the insect *Hox *genes *Ultrabithorax *(*Ubx*) and *Antennapedia *(*Antp*), and implicate the latter in the formation and diversification of novel, butterfly-specific wing patterns.

**Results:**

First, we describe a conserved pattern of *Ubx *expression and a novel pattern of *Antp *expression in wing discs of *Bicyclus anynana *butterflies. The discrete, reiterated domains of Antp contrast with the typical expression of Hox genes in single continuous regions in arthropod embryos. Second, we show that this pattern is associated with the establishment of the organizing centres of eyespots. *Antp *upregulation is the earliest event in organizer development described to date, and in contrast to all genes implicated in eyespot formation, is exclusive to those centres. Third, our comparative analysis of gene expression across nymphalids reveals unexpected differences in organizer determination.

**Conclusions:**

We show that the Antp's recruitment for the formation of novel traits in butterfly wing discs involved the evolution of new expression domains, and is restricted to a particular lineage. This study contributes novel insights into the evolution of *Antp *expression, as well as into the genetic mechanisms underlying morphological diversification. Our results also underscore how a wider representation of morphological and phylogenetic diversity is essential in evolutionary developmental biology.

## Background

The origin and diversification of novel traits is one of the most exciting unresolved issues in evolutionary developmental biology [1-4]. In the past two decades, multiple studies revealed that novelties often evolve through "teaching old genes new tricks", as shared genes and/or gene regulatory networks become co-opted to perform new functions during development (reviewed in [5-8]). Such recruitment can occur via the acquisition of new expression domains, as has been shown for insect appendage patterning genes redeployed for the development of head/pronotum horns in beetles [9], abdominal legs in sepsid flies [10], and wing eyespots in butterflies [11]. Conserved transcription factors can also acquire new target genes within their ancestral expression domains; the diversification of insect wings, for example, has been associated with changes in the set of genes regulated by the Hox protein Ultrabithorax [12-14].

Hox proteins are conserved homeodomain transcription factors that specify segment identity and are expressed in characteristic patterns along the antero-posterior axis of metazoan embryos [15]. For example, Ultrabithorax (Ubx) and Antennapedia (Antp) are crucial for the specification of thoracic segments and are associated with emblematic homeotic transformations of insect appendages [5,15]. Comparative studies of *Hox *genes during embryogenesis revealed that changes in their expression and activity played crucial roles in the evolution of animal body plans [16-18]. In contrast, little is known about their contribution to the formation of lineage-specific traits that develop during post-embryonic stages. Here, we investigated the involvement of Ubx and Antp in the development and diversification of butterfly colour patterns that start to be established in larval wing discs.

Butterfly wing patterns are visually compelling examples of evolutionary innovation. Pattern elements such as stripes, spots, chevrons, and bands are not homologous to pigment patterns in other animals [3], and can play important roles in predator avoidance [19] and/or mate choice [20]. Wing pattern diversity is astounding, with striking variation documented not only between species, but also between different wing surfaces of the same individual [21]. Nevertheless, colour patterns of most butterflies can be recognized as derivations of the "nymphalid groundplan", a schematic representation of homologies among different elements, inferred from their morphology and location on the wing [21,22]. Many butterflies of the family Nymphalidae bear (a series of) marginal eyespots, also called border ocelli, composed of concentric rings of contrasting colours. Even though the morphology of nymphalid eyespots can vary considerably, their location along the wing margin suggests that they have evolved through modification of ancestral marginal bands, which first 'resolved' into spots and later diversified in size and colour [21,22] (but see [23] for an alternative hypothesis). Similarities in the cellular and genetic mechanisms of eyespot formation, revealed in laboratory models *Junonia coenia *and *Bicyclus anynana *(reviewed in [24-26]), further support a common evolutionary origin of these pattern elements in nymphalids.

Several regulatory genes and signalling pathways involved in such conserved processes as embryo segmentation [27,28], appendage formation [11], and wound healing [29] have been recruited for eyespot formation in butterfly wings. Colour rings are presumably induced in a concentration-dependent manner by morphogens produced in the presumptive eyespot centres in pupal wings [30,31]. The establishment of these organizing centres occurs during the last larval instar and involves the Hedgehog (Hh) and Notch (N) signalling pathways and the transcription factors Distal-less (Dll) and Spalt [32-35]. Similar expression of these genes in eyespot organizers of all species examined to date is consistent with the common evolutionary origin of these pattern elements [21,22]. In this study, we report on the recruitment of the *Hox *gene *Antp *in the early establishment of eyespot organizers during post-embryonic development. Our broad comparative analysis across nine species of the family Nymphalidae shows that the involvement of this and other genes in eyespot formation can be very lineage-specific, raising interesting issues about homologies.

## Results and discussion

### A novel expression pattern for *Antp *in the organizers of an evolutionary novelty

**Figure 1:**
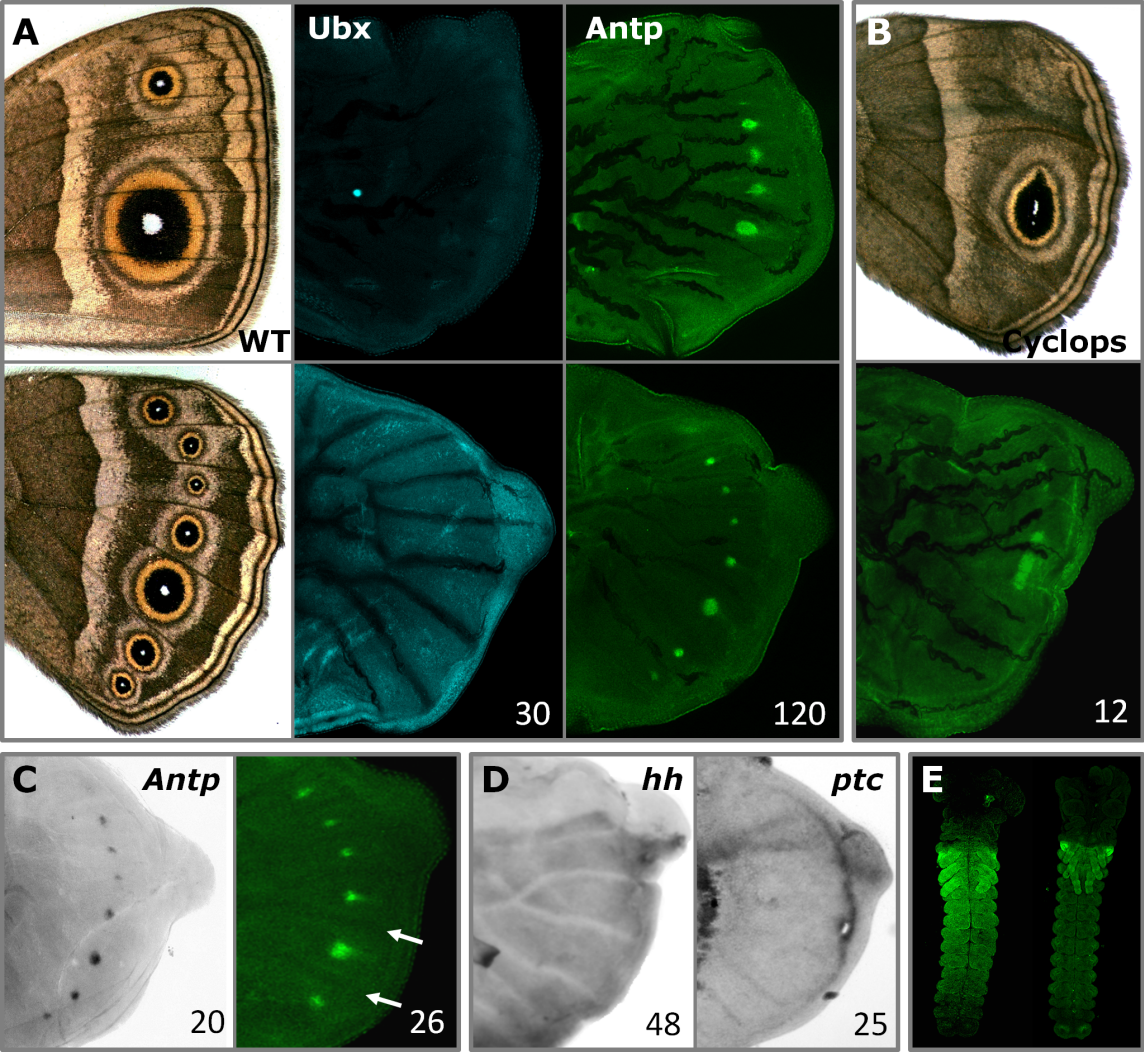
**Gene expression in the eyespot model *Bicyclus anynana *(Nymphalidae, Satyrinae)**. (**A**) Fore-(top) and hindwing (bottom) of 'wild-type' adult and larval wing discs visualized for Ubx (blue) and Antp (green). Ubx is detected throughout the hindwing, but not in the forewing (as is characteristic of insects), and is not associated with any colour pattern element (the bright spot visible in the forewing is an artefact). Antp is detected in the presumptive eyespot organizers in both fore- and hindwing. (**B**) Adult (top) and larval (bottom) hindwing of Cyclops venation mutant with altered eyespot number and shape [28]: *Antp *is upregulated in a single elongated organizer, matching the morphology of the adult eyespot centre. (**C**) Larval hindwings stained for *Antp *mRNA (left) and protein (right). Antp is detected in eyespot centres shortly after the last larval molt, prior to the extension of trachea into the vein lacunae (arrows) and before the upregulation of other organizer proteins (co-stainings of Antp, N and Dll in early wings are shown in Additional File 1). (**D**) Larval hindwings stained for *hh *and *ptc *mRNA. Absence of both transcripts in *B. anynana *eyespot fields (in contrast to *J. coenia *[33]) reveals genetic divergence in organizer determination. (**E**) Immunostainings in embryos of *B. anynana *(left) and *J. coenia *(right) at 30 to 40% development [63] show the typical pattern of Antp in thorax and abdomen. Proteins are shown in colours, mRNA in gray; numbers indicate individuals examined.

Immunostainings in larval wing discs of *Bicyclus anynana *revealed a conserved expression pattern for *Ubx *and a novel expression pattern for *Antp*. In 30 individuals examined, spanning all sequential stages of larval wing development (*cf*. the extent of tracheal extension into vein lacunae [35]), the Ubx protein was detected throughout the entire hindwing and nowhere in the forewing (Figure 1A). The difference between fore- and hindwing is identical to that described in other insects and is consistent with Ubx's role in the specification of the third thoracic segment, including the associated appendages and their characteristics [13-15]. The fact that *Ubx *is expressed ubiquitously on the hindwing with no association to any particular wing regions suggests that this *Hox *gene, for which changes in expression have been associated with colour pattern transformations in *J. coenia *[13], is not involved in the determination of any specific colour pattern element in *B. anynana*.

In contrast to *Ubx*, the *Hox *gene *Antp *was up-regulated in both fore- and hindwings in discrete reiterated domains. These correlate with the position and shape of eyespot organizers in 'wild-type' butterflies (Figure 1A; 140 individuals) as well as in mutants with altered eyespot shape and number (Figure 1B; 12 individuals). Both mRNA (Figure 1C) and protein (Figure 1A) were detected in the seven hindwing and in the four (potential) forewing eyespot organizers (that is, two that typically lead to eyespot formation in 'wild-type' butterflies and two that can, and that do so in laboratory mutants and selection lines [36,37]). Hence, the up-regulated levels of *Antp *in larval wings coincide with the epidermal cells which are competent to induce eyespot formation later on. Furthermore, to our knowledge, such an expression pattern is novel for *Antp*, which was never before detected in the developing insect wing blade (even though it is up-regulated in the margin of imaginal wing discs of *Drosophila *[15]), nor in a series of discrete and reiterated domains. This serially-repeated pattern along the antero-posterior axis of the wing contrasts with the characteristic continuous domain of any *Hox *gene along the antero-posterior axis of developing arthropod embryos (see [15] and Figure 1E). Interestingly, this is also one of the few known examples of *Hox *gene co-option through evolution of a new expression pattern [38-41]. Other *Hox *genes, on the contrary, have been shown to regulate the formation of insect novelties through acquisition of novel targets within their ancestral domains of expression [42].

### *Antp *expression is the earliest event in organizer establishment

Up-regulation of *Antp *occurs shortly after the last larval molt, before that of any other gene so far associated with the establishment of eyespot organizers. The protein was already detected in eyespot centres in 21 out of 26 individuals examined during the first two days of the final instar, before the extension of trachea into the vein lacunae (Figure 1C and Additional file 1). All other previously described organizer genes, for example, *N *(until now the earliest gene detected in eyespot organizers [34]), and *Dll *and *engrailed *(two other early "eyespot genes" [32,33]) are known to be up-regulated in eyespot centres only after the tracheal expansion [35]. Importantly, *Antp *was up-regulated in the presumptive eyespot centres only. This is in contrast to all previously described genes, whose expression in eyespot organizers in larval wing discs is accompanied either by 1) butterfly-specific expression in intervein stripes (*N *and *Dll*), presumably associated with midvein pigment patterns [34], or 2) insect-specific expression along the wing margin (*Dll*) and in the posterior wing compartment (*engrailed*), associated with their conserved function in wing development [32,33].

The finding that *Antp *is expressed exclusively in eyespot organizers suggests that the recruitment of this *Hox *gene is specifically associated with the development of these novelties in larval wing discs. Also, its early expression suggests its involvement in the very initial step of organizer establishment. It will be fascinating to investigate the specific molecular changes underlying the co-option of this *Hox *gene, as well as its interactions with downstream regulators of eyespot development. Future biochemically-focused analysis can establish if and how *Antp *is up-regulated by (yet unknown) signals diffusing from the wing veins and/or margin [21]. It can also investigate if Antp activates *Dll *transcription via N signalling in butterfly wings, a type of interaction that is known to induce ectopic leg formation on *Drosophila *heads [43]. The latter is reminiscent of the idea that eyespots evolved by co-option of the insect appendage patterning network and can be seen as 'flat legs' on butterfly wings [11]. Another exciting issue is whether this regulatory network was co-opted as a whole, or whether genes such as *Antp*, *N *and *Dll *were recruited to eyespot patterning individually and re-wired *de novo *[44].

### Different genes in eyespot organizers of two nymphalid lab models

**Figure 2:**
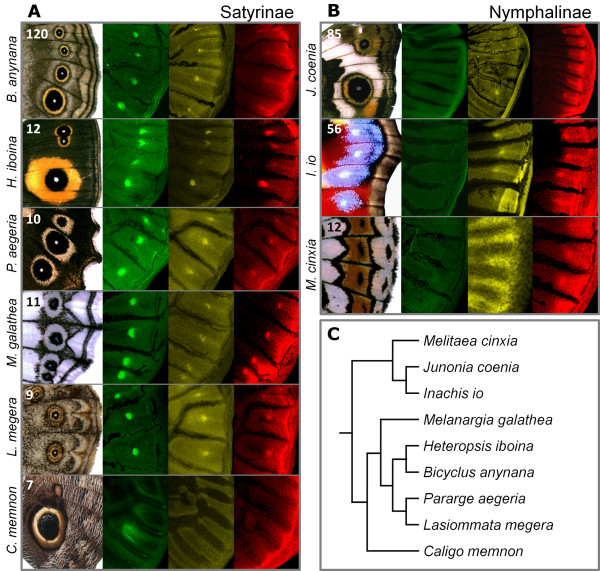
**Genetic divergence in determination of nymphalid eyespot organizers**. (**A**) Localization of Antp (green), N (yellow) and Dll (red) in larval wing discs of representatives of the subfamily Satyrinae. Shown are sections of adult wings and the corresponding sections of larval wing discs at late stages of development, when tracheae are extended into vein lacunae (visible here as black lines). N and Dll are initially expressed in non-organizer areas (both in the intervein stripes, and Dll also along the wing margin), their expression in eyespot organizing centres occurs after extension of the trachea into the lacunae (except in the basal satyrine *C. memnon*). (**B**) Expression of *N *and *Dll *in subfamily Nymphalinae resembles that in Satyrinae, but Antp is absent from eyespot organizers at all stages of larval wing development. In both panels, numbers of individuals used for gene expression analysis in larval wings are shown on adult wing image. (**C**) Phylogenetic relationship among butterflies examined in this study, following [45].

Our analysis of *Antp *expression in *B. anynana *(subfamily Satyrinae) suggested a role in the establishment of eyespot organizers. Next, we investigated whether this *Hox *gene plays a similar role in another nymphalid eyespot model, *Junonia coenia *(subfamily Nymphalinae). Despite the fact that the two species have diverged some 90 MYA [45], their eyespots have strikingly similar appearance: in both species they are formed by a central white pupil, an inner black disc, and an outer golden ring (Figure 2). Like all nymphalid border ocelli, they are thought to have a common evolutionary origin [21]. Surprisingly, our analysis of wing discs from 85 *J. coenia *individuals, covering all stages of the last instar wing development, revealed that, in contrast to what happens in *B. anynana *(Figure 2A), *Antp *is never expressed in eyespot organizers or elsewhere in the wings of *J. coenia *(Figure 2B). Immunostainings in embryos of *B. anynana *and *J. coenia *(Figure 1E) showed typical expression of *Antp *(strong in the thorax and weak in the abdomen [46-48]), confirming that the anti-Antp antibody does recognize the target protein in both species. In contrast to Antp, N and Dll were detected in the intervein midlines and in eyespot organizers in both lab models (Figure 2A, B). These findings suggest that expression of N and Dll in *J. coenia *is under the control of some other factors, and that the regulatory network establishing eyespot organizing centres is not as conserved as previously thought [26]. We, therefore, examined whether other genes, implicated in eyespot formation in *J. coenia*, and assumed to play similar roles in *B. anynana*, have comparable expression patterns in both species.

Previous work implicated Hh signalling, crucial for compartmentalization of insect wings [49], in *J. coenia *eyespot organizer determination [33]. We examined expression patterns of *hh *(the same part of the transcript detected in *J. coenia *eyespots [33]) and its receptor-encoding gene *patched *(*ptc*) in larval wing discs of *B. anynana *(48 and 25 individuals, respectively; Figure 1D). Transcripts of both genes were detected in spatial patterns typical of all insects (that is, *hh *in the posterior wing compartment, *ptc *along the antero-posterior compartment boundary [33,50,51]). However, they were not detected in the eyespot fields of *B. anynana*, even though all stages of the last instar wing development were examined. This suggests that the Hh signalling pathway might not be involved in eyespot organizer determination in *B. anynana*. This absence of the Hh ligand and its receptor Ptc is quite remarkable as it suggests a potentially Hh-independent activation of the signal transducer *cubitus interruptus *and its target *engrailed*, both previously shown to be expressed in *B. anynana *organizers in larval wings [33].

### Broad comparative analysis reveals genetic divergence in organizer establishment

The findings that different genes are expressed in eyespot organizers on wing discs of two lab models are in stark contrast with all previous studies [32-34], and raise questions about the origin and diversification of border ocelli in Nymphalidae. To further address the involvement of *Antp *in the evolutionary diversification of nymphalid (eye)spots, we examined the expression of different organizer genes in representative species from the subfamilies Satyrinae and Nymphalinae (Figure 2).

Our analysis in four more species of the tribe Satyrini (subfamily Satyrinae [45]), revealed that *Antp*, *N*, and *Dll *are expressed in the presumptive eyespot organizers, similar to *B. anynana *which also belongs to this tribe (Figure 2A). In the more basal *Caligo memnon *(tribe Brassolini, subfamily Satyrinae [45]) (Figure 2C), only Antp was detected in the presumptive organizers. In this species, N and Dll were not detected in the organizer regions but were still present in other cells of the developing wing: N throughout the wing, Dll along the wing margin. The absence of these two proteins in *Caligo *eyespots and intervein areas (contradicting a recent suggestion that expression of these genes in the intervein midline is common to all butterflies [34]) might be associated with their unusual morphology (lack of round white centres) or with their unusual location (hindwing eyespots are shifted proximally). We also analyzed two additional species of the subfamily Nymphalinae, to which *J. coenia *belongs [45]. Representatives of this subfamily exhibit a wide range of marginal patterns (Figure 2B), including eyespots (as in *J. coenia*) or spots with no obvious concentric rings (as in *Inachis io *and *Melitaea cinxia*). Immunostainings revealed that expression of *N *and *Dll*, but not of *Antp*, is associated with the establishment of these pattern elements in larval wing discs of Nymphalinae.

The diversification of nymphalid eyespot morphology had been previously attributed to changes in gene interactions during the pupal stage [27], with the same transcription factors associated to different eyespot rings and/or pigments in different species [52]. In contrast, similarities in the inductive properties of, and genes expressed in, the eyespot organizers of *J. coenia *and *B. anynana *[30-34] supported the idea that organizer determination in the larval stage is conserved, reflecting their common evolutionary origin. However, our analysis of gene expression in nine butterflies from the family Nymphalidae revealed unexpected differences in the earliest known step of pattern formation. We found that Antp and Hh signalling are associated with eyespot organizers in Satyrinae and Nymphalinae, respectively, while N and Dll are present in the intervein midline and border ocelli in both subfamilies, with *C. memnon *as an exception (Figure 2A). Taken together, our findings suggest that the genetic mechanisms underlying the first known step of eyespot formation differ substantially across nymphalids. This can be either because of their independent evolutionary origin, or because of later divergence and great flexibility in gain and loss of some components of the eyespot regulatory network.

## Conclusions

Our study describes a novel expression pattern for the *Hox *gene *Antp *in butterfly wings, associated with the initial differentiation stage of an evolutionarily novel trait. We show that *Antp *is upregulated exclusively and before any other described gene in the presumptive eyespot organizers, but only in one of two butterfly lineages examined. Altogether, our results show that Antp was redeployed to the developing wing disc and is associated with the target novel trait (and is probably involved in its formation), but only in a particular lineage (that is, it is associated with its diversification). Co-option of this conserved embryonic patterning gene to eyespot formation in larval wings occurred through the acquisition of a new expression pattern - a series of discrete domains that contrast with the characteristic *Hox *gene expression in contiguous broad domains in arthropod embryos. This illustrates that key developmental genes can evolve novel expression characteristics, and opens up more biochemically-centred questions, including the identification and characterization of the molecular factors immediately up- and downstream of *Antp *in this novel context.

We also show that the expression patterns of *Ubx*, *hh *and *ptc *in larval wing discs of *B. anynana *are consistent with their conserved roles in all insects and do not correlate with any particular colour patterns in this species. This is particularly interesting for *ptc *and *hh *which have been associated with eyespot formation in another eyespot model and are not expressed in *B. anynana *eyespots even though their signal transducer *cubitus interruptus *and downstream target *engrailed *are [33].

Our comparative analysis across nine species of Nymphalidae further highlights the genetic divergence in the first step of organizer determination and demonstrates that the redeployment of conserved genes can be very lineage-specific. This raises interesting issues about the identification of homologies. Homology is a concept with a clear definition (common ancestry) but of notoriously difficult assessment (see [3,53-55]). Homologies among structures or patterns are typically inferred from their morphology and location, and are further confirmed by comparative studies of the underlying developmental programmes and associated genetic networks [52,56]. However, homologous structures can be determined by (more or less) diverged genetic machinery, as are segments of insects [57]. Conversely, non-homologous structures can share genetic and developmental mechanisms, as is the case for beetle horns and insect legs [9]. Nymphalid eyespots, with well studied serial homologies across elements repeated on one wing and homologies across species [21-23], offer a good opportunity to investigate the extent to which morphological and genetic data are consistent in the assessment of common evolutionary origin. Our broad comparative analysis revealed the genetic divergence of the early stages of eyespot organizer establishment, which might suggest independent evolution of eyespots in different lineages. This study emphasizes how a wider representation of both morphological and phylogenetic diversity is so crucial in evolutionary developmental biology [58,59].

## Methods

### Experimental animals

*Bicyclus anynana *wild-type and *Cyclops *laboratory stocks were reared as in [60]. Larvae of other species were purchased from butterfly houses or provided by colleagues, and reared in climate rooms or at room temperature indoors and fed on *Oplismenus *(*Heteropsis iboina*) or *Poa *grasses (*Pararge aegeria *and *Melanargia galathea*), wheat (*Lasiommata megera*), narrowleaf plantain (*Junonia coenia *and *Melitaea cinxia*), nettles (*Inachis io*), or banana leaves (*Caligo memnon*).

### Gene cloning in *B. anynana*

Total RNA was extracted from embryos and larval wings with Trizol (Invitrogen, Paisley, UK) and treated with DNase (Ambion, Nieuwerkerk a/d lJssel, The Netherlands). The first strand complementary DNA was prepared using Reverse Transcription System (Promega, Leiden, the Netherlands) and the SMARTer RACE kit (Clontech, Saint-Germain-en-Laye, France) A 159 base pairs (bp) fragment of *Antp *was amplified from embryonic cDNA with degenerate primers 5'-CAGACCCTGGAGCTGGAGAARGARTTYCAYT and 5'-GCCCTTGGTCTTGTTCTCCTTYTTCCAYTTC, and extended with the 5'RACE in two rounds using primers 5'-GATTTGGCGCTCGGTGAGACAGAGG and 5'-CCGCGTCAGGTATCGGTTGAAGTGG. Sequence analysis of the obtained 450 bp fragment of *Antp *revealed that this partial cds encodes 150 amino acids and shares 95% amino acid identity with the Antp protein of the reference lepidopteran, the silkworm *Bombyx mori *(Additional file 2). A 339 bp sequence of *hh *(provided by Arjen van't Hof) was extended to 548 bp with the 5'RACE using 5'-GCTCCAGTGCCCACTGATGATTCTG and 5'-ACACTGATGGCGAGCGTGTTCAACT primers. The corresponding 182 amino acid product is closely similar to Hh proteins of other insects, and shares 93% identity with Hh of *J. coenia *(Additional file 2). A 2,305 bp sequence of *ptc *was provided by Arjen van't Hof. The corresponding 744 amino acid product shares 97% identity with Ptc of *J. coenia *(Additional file 2). All sequences were edited in BioEdit and aligned against their insect homologues in NCBI BLAST, conserved domains were detected with CD-search option. ExPASy's translation tool [61] was used to obtain the translations of the nucleotide sequences. The ClustalW2 multiple alignment tool [62] was used to produce the protein alignments and trees (with default settings). The nucleotide sequences of *B. anynana Antp*, *hh *and *ptc *have been deposited to GenBank (respective accession numbers:HQ020406, HQ020407, HQ020408).

### *In situ *hybridizations and immunohistochemistry

During the final larval instar, butterfly wing discs develop the characteristic venation pattern accompanied by the extension of trachea into vein lacunae, which was used for staging of wing disc development (cf. [35]). Stainings of embryos staged according to the system developed for *M. sexta *[63] and larval wings were performed as described in [60]. The monoclonal mouse anti-Antp 4C3 [64] (dilution 1:50) and anti-N C17.9C6 [65] (1:5) were obtained from the Developmental Studies Hybridoma Bank; the polyclonal rabbit anti-Dll [66] (1:200) and the monoclonal mouse anti-Ubx/Abd-A FP6.87 [67] (1:10) were provided by Sean B. Carroll. Alexa Fluor 488 anti-mouse and Texas Red anti-rabbit (Molecular Probes, Invitrogen) were used as secondary antibodies (1:200). Right fore- and hindwing were stained with anti-Antp antibody, left fore- and hindwing of same individual were stained with anti-N and anti-Dll antibodies. Whole mount *in situ *hybridizations were performed with digoxigenin-labeled antisense and sense (control) probes, detected with NBT/BCIP (Roche, Almere, The Netherlands) Images were collected on a Zeiss Imager M1 laser, Sliedrecht, The Netherlands) scanning confocal microscope, or with a Leica DC200 digital camera on a Leica MZ125 microscope.

## Abbreviations

Antp: Antennapedia; Dll: Distal-less; Hh: Hedgehog; N: Notch; Ptc: Patched; Ubx: Ultrabithorax.

## Competing interests

The authors declare that they have no competing interests.

## Authors' contributions

SVS and PB conceived and designed the study, analyzed the results and wrote the manuscript. SVS collected the data on Antp, N and Dll. SVS and MSPM collected the data on Hh and Ptc. All authors read and approved the final manuscript.

## Supplementary Material

Additional file 1 **Early gene expression in *Bicyclus anynana *larval wing discs**. Forewings of one single individual at early last instar stage, prior to extension of trachea into the vein lacunae. Right wing disc stained for detection of Antp protein, and left wing disc for N and Dll proteins (see Material and methods). The right wing disc is also shown in bright field. Antp is already detected in the four putative eyespot organizers at this stage, while N and Dll are not. N is expressed throughout the wing, and Dll in the wing margin and intervein stripes. Of the 26 early last instar individuals examined, 21 had this exact pattern (with only Antp in the organizers), and five had none of the three genes yet detectable in eyespot centers. Click here for file

Additional file 2 **Alignments and phylogenetic trees of insect Antp, Hh and Ptc proteins**. (**A**) Schematic representation of the phylogenetic relationship between insect species (adapted from the Tree of Life [68]) for which protein sequences are compared with those encoded by the *B. anynana *genes cloned in this study. Multiple sequence alignment of the predicted *B. anynana *proteins with the corresponding fragments of orthologous proteins from other insects and the consensus tree that was generated from it [60] for: (**B**) Antp (grey box corresponds to the homeobox domain, *cf*. [69]), (**C**) Hh (blue box corresponds to the amino-terminal signalling domain, *cf*. [69]) and (**D**) Ptc (pink box corresponds to the Sterol-sensing domain of SREBP cleavage-activation, *cf*. [69]). In all alignments the numbers on the right correspond to amino acid number; sequence identities are marked with (*), conserved substitutions with (:) and semi-conserved substitutions with (.), *cf*. ClustalW [62]. Gray underlining indicates parts of the sequences that were used as probes for *in situ *hybridization experiments. GenBank accession numbers of all protein sequences are shown at the end of tree branches. Click here for file
