# Supplementary figures and images for: Involvement of the conserved Hox gene Antennapedia in the development and evolution of a novel trait
Source: EvoDevo. 2011 Apr 19;2:9. doi: 10.1186/2041-9139-2-9 (PMC3108338; doi:10.1186/2041-9139-2-9)

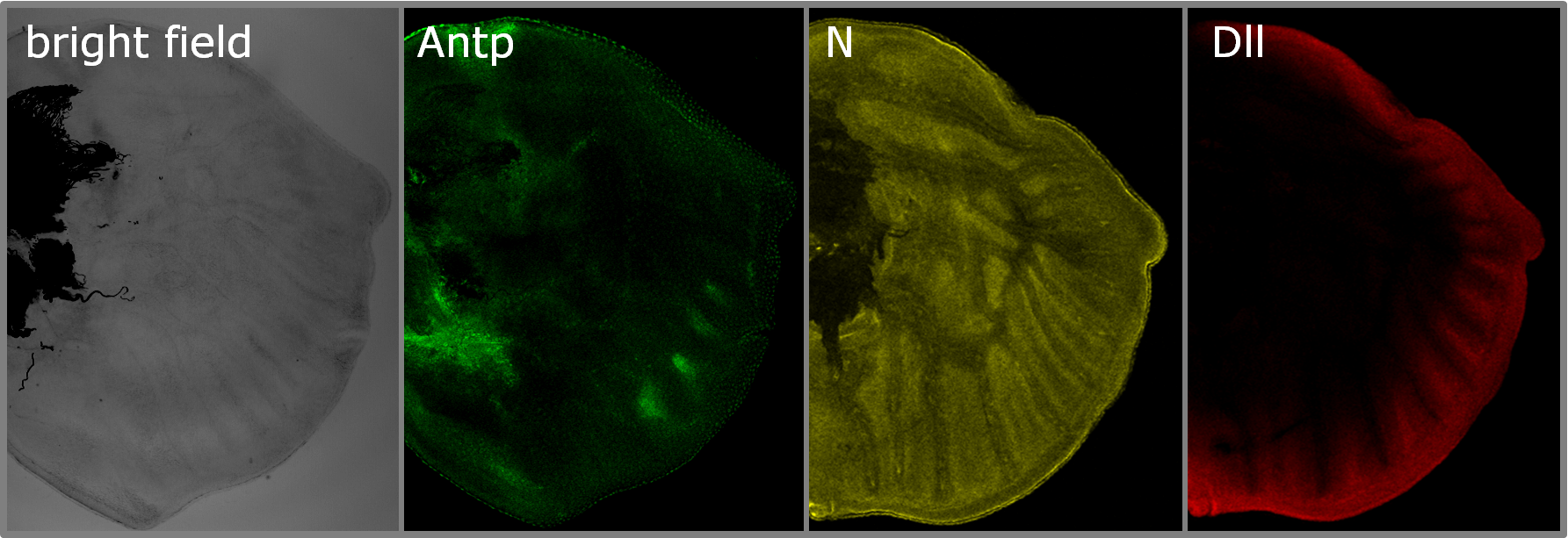

Supplement: Additional file 1 — Early gene expression in Bicyclus anynana larval wing discs. Forewings of one single individual at early last instar stage, prior to extension of trachea into the vein lacunae. Right wing disc stained for detection of Antp protein, and left wing disc for N and Dll proteins (see Material and methods). The right wing disc is also shown in bright field. Antp is already detected in the four putative eyespot organizers at this stage, while N and Dll are not. N is expressed throughout the wing, and Dll in the wing margin and intervein stripes. Of the 26 early last instar individuals examined, 21 had this exact pattern (with only Antp in the organizers), and five had none of the three genes yet detectable in eyespot centers. [file 2041-9139-2-9-S1.TIFF]
