# Supplementary material for: Involvement of the conserved Hox gene Antennapedia in the development and evolution of a novel trait
Source: EvoDevo. 2011 Apr 19;2:9. doi: 10.1186/2041-9139-2-9 (PMC3108338; doi:10.1186/2041-9139-2-9)
Supplement: Additional file 2 — Alignments and phylogenetic trees of insect Antp, Hh and Ptc proteins. (A) Schematic representation of the phylogenetic relationship between insect species (adapted from the Tree of Life [68]) for which protein sequences are compared with those encoded by the B. anynana genes cloned in this study. Multiple sequence alignment of the predicted B. anynana proteins with the corresponding fragments of orthologous proteins from other insects and the consensus tree that was generated from it [60] for: (B) Antp (grey box corresponds to the homeobox domain, cf. [69]), (C) Hh (blue box corresponds to the amino-terminal signalling domain, cf. [69]) and (D) Ptc (pink box corresponds to the Sterol-sensing domain of SREBP cleavage-activation, cf. [69]). In all alignments the numbers on the right correspond to amino acid number; sequence identities are marked with (*), conserved substitutions with (:) and semi-conserved substitutions with (.), cf. ClustalW [62]. Gray underlining indicates parts of the sequences that were used as probes for in situ hybridization experiments. GenBank accession numbers of all protein sequences are shown at the end of tree branches. [file 2041-9139-2-9-S2.PDF]

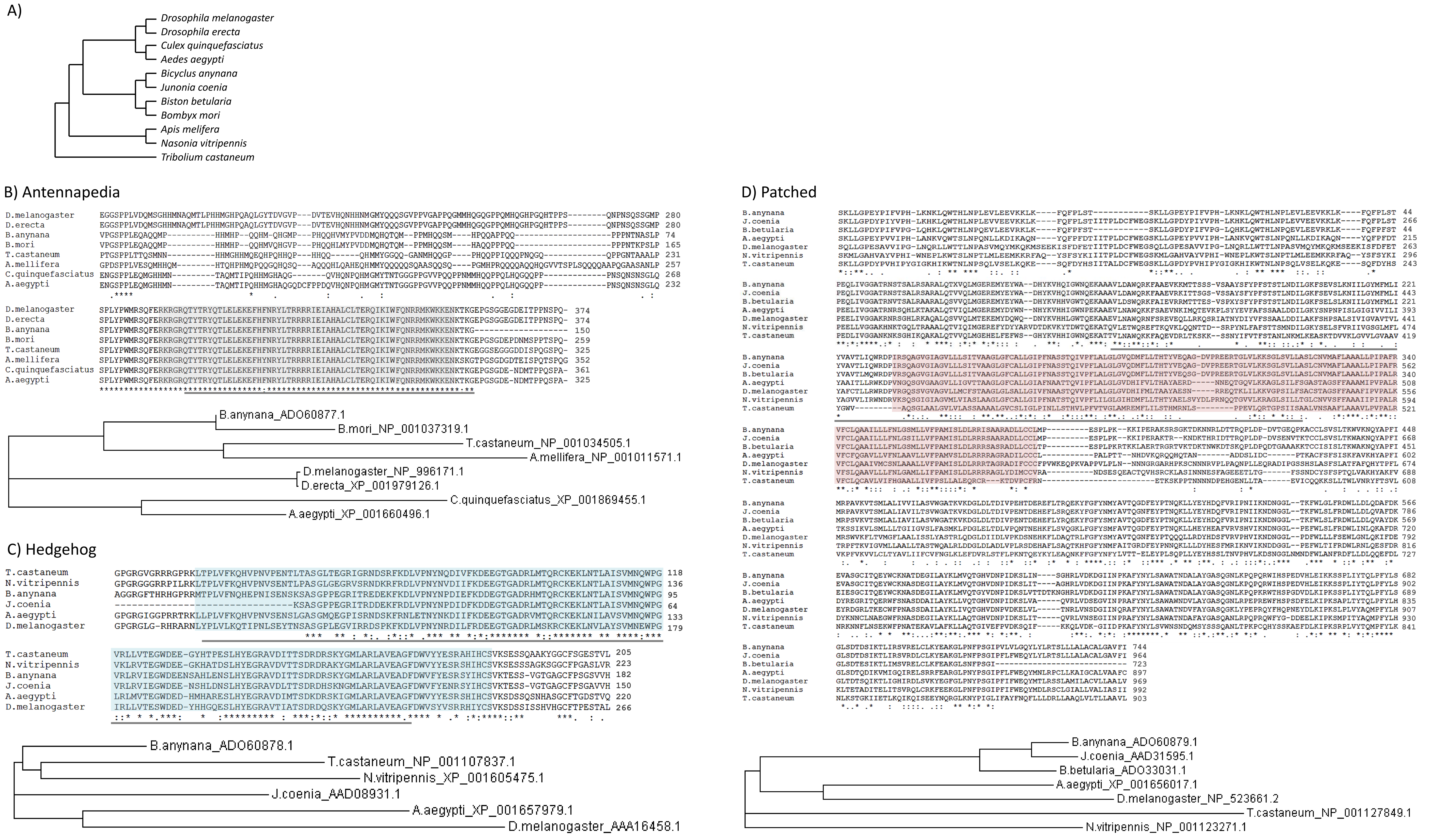
A)
Drosophila melanogaster
Drosophila erecta
Culex quinquefasciatus
Aedes aegypti
Bicyclus anynana
Junonia coenia
Biston betularia
Bombyx mori
Apis melifera
Nasonia vitripennis
Tribolium castaneum
B) Antennapedia
B.anynana_ADO60877.1
B.mori_NP_001037319.1
T.castaneum_NP_001034505.1
A.mellifera_NP_001011571.1
D.melanogaster_NP_996171.1
D.erecta_XP_001979126.1
C.quinquefasciatus_XP_001869455.1
A.aegypti_XP_001660496.1
C) Hedgehog
B.anynana_ADO60878.1
T.castaneum_NP_001107837.1
N.vitripennis_XP_001605475.1
J.coenia_AAD08931.1
A.aegypti_XP_001657979.1
D.melanogaster_AAA16458.1
D) Patched
B.anynana_ADO60879.1
J.coenia_AAD31595.1
B.betularia_ADO33031.1
A.aegypti_XP_001656017.1
D.melanogaster_NP_523661.2
T.castaneum_NP_001127849.1
N.vitripennis_NP_001123271.1
